# Draft Genome Sequence of *Pseudomonas syringae* PDD-32b-74, a Model Strain for Ice-Nucleation Studies in the Atmosphere

**DOI:** 10.1128/genomeA.00742-17

**Published:** 2017-07-27

**Authors:** L. Besaury, P. Amato, M. Sancelme, A. M. Delort

**Affiliations:** CNRS, Institut de Chimie de Clermont-Ferrand, Université Clermont Auvergne, Clermont-Ferrand, France

## Abstract

We report here the whole genome sequence of *Pseudomonas syringae* PDD-32b-74, a gammaproteobacterium isolated from cloud water. This microorganism is equipped with ice-nucleation protein and biosurfactant genes that could potentially be involved in physicochemical processes in the atmosphere and clouds.

## GENOME ANNOUNCEMENT

Atmospheric microorganisms can play a role in numerous atmospheric physicochemical processes, including cloud droplet formation, ice nucleation, and precipitation initiation (1–4). Among those microorganisms isolated from cloud water, bacteria affiliated with the genus *Pseudomonas* are the most abundant and frequent (5). *P. syringae* PDD-32b-74 was recovered by the culture of cloud water sampled in November 2009 from the meteorological station on Puy de Dôme Mountain in France (altitude of 1,465 m) on R2A agar medium incubated at 17°C under aerobic conditions. Once purified from the original colony, the strain was identified based on its 16S rRNA gene sequence (GenBank accession no. HQ256872). *P. syringae* PDD-32b-74 has the ability to produce siderophores to satisfy its iron requirements (6); it thus potentially interferes with cloud chemical reactivity. This strain has been found to be highly ice-nucleation positive (7, 8) and to produce biosurfactants known for facilitating its spread on surfaces (9). We report here the draft genome sequence of *P. syringae* PDD-32b-74 to allow for further investigation into the role of this bacterium in clouds, as well as its capacity to survive and adapt to atmospheric stresses. 

Whole-genome shotgun sequencing (2 × 150 bp) was prepared using the Nextera DNA sample preparation kit (Illumina, San Diego, CA, USA) following the manufacturer’s user guide and sequenced on the Illumina MiSeq platform (MR DNA [Molecular Research] Shallowater, TX, USA). Sequence data files were filtered for quality using FastQC, trimmed using Prinseq-Lite (10), and then *de novo* assembled with SPAdes (11). A total of 49 contigs were generated with an average coverage of 22.6-fold. The average contig size was 116,408 bp, and the *N*_50_ contig size was 288,783 bp. The size of the assembled genome is 5,704,000 bp with a GC content of 59.1%, which is within the range of known values for *Pseudomonas* genomes (12).

The draft genome of *P. syringae* PDD-32b-74 was annotated using the RAST annotation server (http://rast.nmpdr.org). The genome contains 65 RNAs, 4 rRNAs, 57 tRNAs, and 4,934 protein-coding genes, of which 50% were assigned to a total of 520 SEED subsystems. Among these SEED subsystems, 181 were affiliated to stress response (79 to oxidative stress, 32 to osmotic stress, 5 to cold shock, 17 to heat shock, 29 detoxification, and the rest were unclassified). As expected from its phenotype, one ice-nucleation protein (INA_Z; 1,259 amino acids long) was detected in the genome content of *P. syringae* PDD-32b-74, as well as four protein-coding genes associated with dormancy and sporulation, two affiliated with the persistence of cells in the environment, and two associated with the spore core dehydration and sporulation processes. All of these could contribute to the survival of *P. syringae* PDD-32b-74 and influence some of the physicochemical processes happening in clouds.

### Accession number(s).

This whole-genome shotgun project has been deposited in GenBank under accession number MTSA00000000.
